# In This Issue

**Published:** 1996

## ALCOHOL RESEARCH AND SOCIAL POLICY

Alcohol sales and consumption are regulated for economic, health, and social purposes. Choosing among potentially conflicting policies can be a daunting task. Dr. Enoch Gordis explains how science can facilitate decisionmaking. Science can play a decisive role in policy development when public support already exists, as in the case of the passage in 1984 of the Federal Uniform Drinking Age Act. Science also can help assess a policy after it has been implemented. An example of this role is the scientific evaluation of the health warning labels on alcoholic beverage containers. Finally, science can investigate the short- and long-term benefits and risks of potential policies. The current scientific examination of the tradeoffs involved in moderate alcohol drinking over the life span is an example of this type of scientific participation in the policy process. (pp. 208–212)

## THE MINIMUM LEGAL DRINKING AGE

Alcohol use among youth is a factor in many public health problems, including traffic crashes, drownings, vandalism, assaults, homicides, suicides, teenage pregnancies, and sexually transmitted diseases. Minimum legal drinking age (MLDA) laws are an example of how scientific research can form the basis for effective public policies. Dr. Traci L. Toomey, Carolyn Rosenfeld, and Dr. Alexander C. Wagenaar describe research findings demonstrating the effectiveness of a higher MLDA in preventing injuries and deaths among youth. The authors suggest ways in which the MLDA can be made even more effective, primarily through increased enforcement and deterrence efforts. (pp. 213–218)

## PREVENTION OF DRINKING AND DRIVING

Alcohol-related traffic deaths among drivers age 21 and older increased between 1994 and 1995, the first increase in 10 years. Dr. Ralph Hingson discusses major legal and community initiatives to reduce the problem of drinking and driving and examines potential measures for further reductions. Legislative initiatives, such as the uniform minimum legal drinking age of 21, administrative license revocation, and lower legal blood alcohol limits for youth and adults, have produced significant declines in alcohol-related traffic deaths. According to Dr. Hingson, ongoing education, improved enforcement, and comprehensive community programs can reduce alcohol-related traffic deaths even further. (pp. 219–226)

## PERSPECTIVES ON ALCOHOL TAXATION

Controversy over alcohol taxation has existed since the Nation’s infancy and has been debated from multiple points of view. Drs. Donald Kenkel and Willard Manning examine the issue of alcohol taxation from each of the following perspectives: public health, revenue generation, economic efficiency, fairness, and effects on employment. The authors note that widely different conclusions about the appropriateness or effectiveness of an alcohol tax can be reached, depending on which perspective is taken. Drs. Kenkel and Manning review research elucidating how various subgroups of drinkers respond to tax-induced higher prices for alcoholic beverages. Such information can assist policymakers trying to balance the trade-offs involved in any proposed tax increase. (pp. 230–238)

## HARM REDUCTION

Although harm-reduction strategies originally developed in the drug prevention field, the concept also has been applied to alcohol abuse, where these approaches aim to reduce the consequences of intoxication without necessarily reducing the level of alcohol consumption. In this article, Dr. Eric Single provides examples of innovative harm-reduction strategies that are proving useful in reducing certain alcohol-related problems. These approaches, which range from the use of special glassware to server training, frequently focus on minimizing the negative consequences of heavy-drinking occasions. Harm reduction may be useful, particularly in light of analyses indicating that the number of heavy-drinking occasions more strongly predicts alcohol problems than does overall consumption level. (pp. 239–243)

## ACCESS TO ALCOHOL

The availability of alcohol, measured in terms of the number of alcohol sales outlets in a given geographic location, is linked to specific patterns of alcohol-related motor vehicle crashes in communities. To curb alcohol-related problems such as violence, traffic crashes, and drinking and driving, community advocates often focus on reducing alcohol availability through modifications in zoning laws and licensing requirements. Drs. Paul J. Gruenewald, Alexander B. Millar, and Peter Roeper describe how research on the relationship between availability and alcohol-related problems can be used to drive policy decisions. The authors review recent research on the effects of outlet density using an adequate community “biogeography” describing the interrelationships among drinkers, their drinking environments, the locations of alcohol outlets, and evidence of alcohol problems. (pp. 244–251)

